# Eclectic Magazine

**Published:** 1874-02

**Authors:** 


ECLECTIC MAGAZINE.—This favorite magazine keeps pace with the literature of the day. It gathers for its readers the cream of foreign literature, carfully and well selected. Every thing in science, art, literature, invention and discovery, is drawn to its pages and its numbers, well preserved, will form a library for instruction, recreation and pleasure to the home circle. Price $5.00. P. Pelton, 108 Fulton street, New York.

